# Selection for Growth and Precocity Alters Muscle Metabolism in Nellore Cattle

**DOI:** 10.3390/metabo10020058

**Published:** 2020-02-06

**Authors:** Nara Regina Brandão Cônsolo, Juliana da Silva, Vicente Luiz Macedo Buarque, Angel Higuera-Padilla, Luis Carlos Garibaldi Simon Barbosa, Andressa Zawadzki, Luis Alberto Colnago, Arlindo Saran Netto, David Edwin Gerrard, Saulo Luz Silva

**Affiliations:** 1 Department of Animal Science, University of São Paulo, Duque de Caxias Norte 225, Pirassununga 13635-900, Brazil; ju-silva@usp.br (J.d.S.); vicentebuarque@usp.br (V.L.M.B.); sauloluz@usp.br (S.L.S.); 2 EMBRAPA Instrumentação, XV de Novembro 1452, São Carlos 13560-970, Brazil; angelruben@hotmail.com (A.H.-P.); garibavet@yahoo.com.br (L.C.G.S.B.); luiz.colnago@embrapa.br (L.A.C.); 3 Department of Food Science, University of Copenhagen, Rolighedsvej 26, 1958 Frederiksberg, Denmark; 4 Department of Animal and Poultry Sciences, Virginia Tech, Blacksburg, VA 24061, USA; dgerrard@vt.edu

**Keywords:** genetic selection, growth, metabolomics, muscle metabolites, NMR spectroscopy, Nellore, precocity

## Abstract

To clarify the relationship between beef genetic selection for growth and precocity with muscle metabolism and metabolites, we performed metabolomic analysis using *Longissimus lumborum* (LL) muscle from Nellore cattle with divergent selection for these traits (high growth, HG; low growth, LG; high precocity, HP; low precocity, LP). Genetic potential for growth affected muscle protein and energetic metabolism. HG animals had a high concentration of arginine, carnosine, and leucine compared to LG animals. HP animals presented a high concentration of glutamine, betaine, creatinine, isoleucine, carnitine, acetyl carnitine, and lower levels of glucose compared to LP animals, affecting protein and fatty acid metabolism. Intensity of selection (high or low) was correlated with changes in protein metabolism, and the type of selection (growth or precocity) affected fat metabolism. In conclusion, both HG and HP appear to be correlated with a high concentration of protein metabolites and changes in protein metabolic pathways, while selection for precocity is more correlated with changes in fat metabolism compared to animals selected for growth.

## 1. Introduction 

Growth is one of the most important selection traits in livestock production. The most common way of measuring growth is by measuring body weight or body weight increase rates in a certain period of time [1]. Growth measurements taken at different ages (i.e., birth, weaning, yearling) as well as sexual maturity, lean meat yield, and meat quality traits have been used as a basis for the selection and improvement of beef cattle worldwide through the expected progeny differences (EPDs) tools. However, selection based on weight and growth rates often leads to late-maturing cattle with high maintenance requirements due to correlated responses [2], which are highly undesirable traits in pasture productions systems. Therefore, sexual maturity or precocity traits are often used for selection purposes in beef cattle [3].

Improvements in average daily gains, body weight, muscle mass, fat deposition, and carcass traits have been reported using genetic merit for growth and precocity as criteria [4,5,6,7]. However, little is known about how the genetic potential for these traits affect muscle metabolism. Given growth and precocity are under strong endocrinological control, investigation of muscle metabolites may prove useful in improving our understanding of how genetic selection changes muscle growth and precocity [8].

Meat metabolites can be assessed by standard one-dimensional (1D) proton nuclear magnetic resonance (^1^H-NMR) spectroscopy, which can quickly characterize and quantify low molecular weight metabolites requiring simple and fast sample processing [9]. Metabolites are effectively the end products of complex reactions occurring in response to genomic and environment control. Therefore, metabolites are sensitive indicators of the genomic influence on a tissue [10]. Additionally, metabolomics can be an important tool to understand muscle metabolism, since some metabolites contribute to various metabolic pathways including muscle hypertrophy, fat deposition, protein degradation, and gustatory sensations [11]. Therefore, the present study hypothesizes that genetic selection for growth and precocity affects an animal’s metabolism and metabolites, improving the understanding of metabolism according to the genetic potential of those traits, and establishing bio-markers that can be used with genome information to improve genetic selection in cattle. The aim of this study was to evaluate the effects of divergent growth and sexual maturity selection on muscle metabolite profiles from Nellore cattle using ^1^H-NMR spectroscopy.

## 2. Results

**Figure 1:**
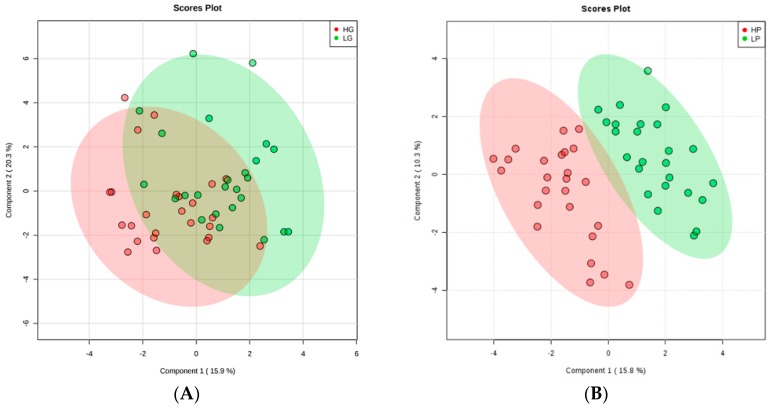
Partial least squares discriminant analysis (PLS-DA) obtained from employing the metabolic concentration of all metabolites detected in the muscle tissue of animals according to: (**A**) Growth: high growth (HG) and low growth (LG) animals are represented by the red and green colors, respectively; and (**B**) Precocity: high precocity (HP) and low precocity (LP) animals represented by the red and green colors, respectively. The validation analysis for growth resulted in the R2 = 0.427, Q2 = 0.105, and Accuracy = 0.64 (Figure S1: Validation statistics for PLS-DA on animals selected for growth); for precocity, R2 = 0.795, Q2 = 0.523, and Accuracy = 0.92 (Figure S2: Validation statistics for PLS-DA on animals selected for precocity).

Thirty-one metabolites were quantified in the ^1^H-NMR spectrum including essential and non-essential amino acids, small peptides, amino acid derivatives, metabolites from glycolysis and the citric acid cycle, organic acids, nucleic acids, sugars, vitamins, cellular antioxidants, metabolites from muscle and fat metabolism, and some other compounds (descriptive analysis; Table S1). Partial least squares discriminant analysis (PLS-DA) was used to visualize the differences in metabolite profiles between the groups. The Component 1/Component 2 scores plot (Figure 1A,B) shows no separation between high growth (HG) and low growth (LG) animals, suggesting no difference in muscle metabolite profiles between them. On the other hand, separation was found between the high precocity (HP) and low precocity (LP) animals, according to the PLS-DA score plot, indicating that their metabolite profiles were different.

Due to the poor separation overall of genetic potential for growth using PLS-DA, the asymptotic general independence test and two-sample Kolmogorov–Smirnov tests were performed to investigate the metabolites that differed between the post-weaning growth groups (Table 1). From both tests, the model showed that arginine, carnosine, and leucine were the most important metabolites separating the HG and LG animals, presenting a higher concentration in HG animals (*p*-values 0.0006861, 0.002277, and 0.0317, respectively for the asymptotic general independence test; and p-values of 0.002318 (D = 0.52), 0.03663 (D = 0.4), and 0.03663 (D = 0.4), respectively, for the two-sample Kolmogoro–-Smirnov test).

**Figure 2:**
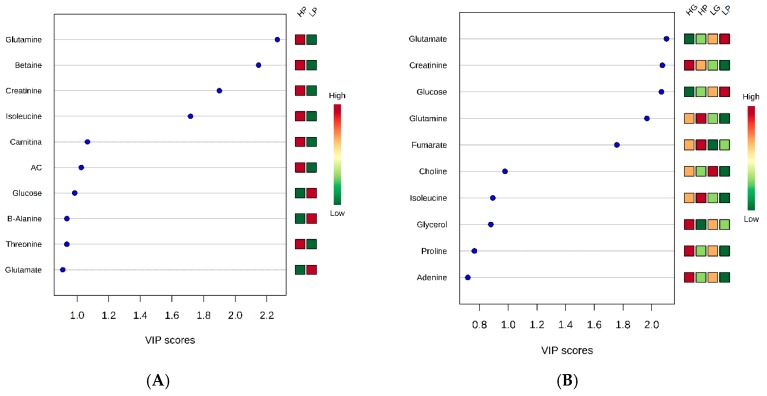
Variable importance in projection (VIP) plot value analyses obtained from (**A**) high precocity (HP) and low precocity (LP) animals; and (**B**) high growth (HG), high precocity (HP), low growth (LG), and low precocity (LP) animals. AC: Acetylcarnitine.

**Figure 3:**
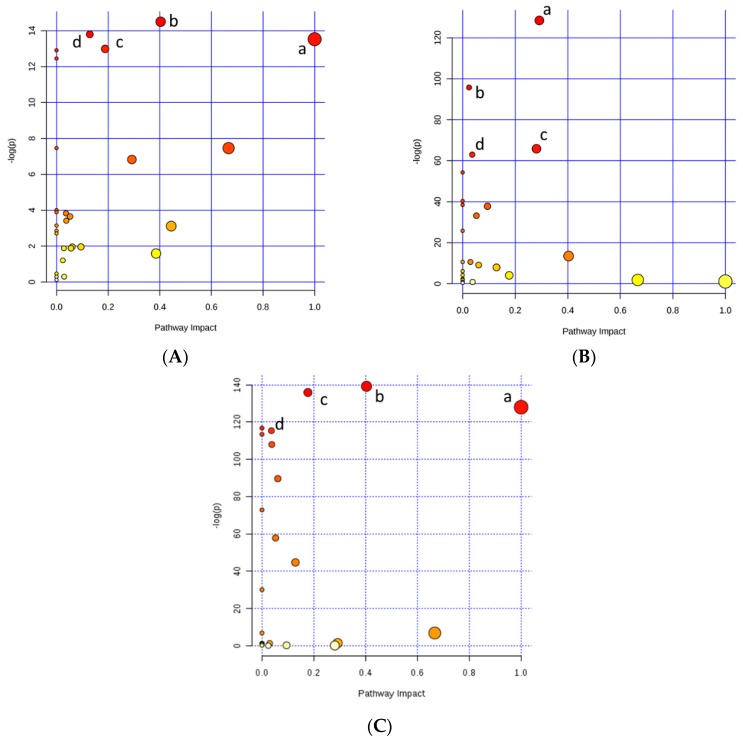
Metabolomics pathways of the meat extract revealed significant differences in the drawn pathway according to (**A**) Precocity: high precocity (HP) and low precocity (LP); (**B**) Selection traits: Growth and Precocity; and (**C**) Intensity of selection: High and Low. In the scatter plot, the x-axis indicates the impact on the pathway whereas the y-axis indicates significant changes in the pathway by detected metabolites. Darker color and larger size represent higher *p*-values from the enrichment analysis and greater impact from the pathway topology analysis, respectively.

Regarding the genetic potential for precocity, as the PLS-DA presented a good separation between groups, the variable importance in the projection (VIP) plot was performed. Differences between HP and LP were found for glutamine, betaine, creatinine, isoleucine, carnitine, acetylcarnitine (AC), and glucose levels (Figure 2A), where only glucose presented a lower concentration for the HG compared to the LG group. The muscle metabolomics pathway affected by precocity were glutamine and glutamate metabolisms (a), alanine, aspartate, and glutamate metabolisms (b), arginine and proline metabolisms (c), and purine metabolism (d) (Figure 3A and Table 2).

Metabolites correlated with protein metabolism such as glutamate, glucose, creatinine, glutamine, fumarate, and isoleucine followed the same pattern for HG and HP and for LG and LP animals (Figure 2B), suggesting that the intensity of selection drives the protein metabolism. The HG and HP groups presented lower amounts of glutamate and glucose and greater concentrations of creatinine, glutamine, fumarate, and isoleucine when compared to the LG and LP groups. On the other hand, metabolites correlated with fat metabolism such as choline and glycerol, showed opposite behavior for HG and HP and similar behavior for HG and LG, and HP and LP, where both metabolites were in greater concentration in the growth potential (GP) group than in the precocity (P) group, suggesting that the selection trait may drive fat metabolism.

In addition, the relevant pathways accessed by selection traits (growth and sexual maturity; Figure 3B and Table 2) were generally more correlated with fat metabolism such as glycine, serine, and threonine metabolisms (a), glycerolipid metabolism (b), glycerophospholipid metabolism (c), and galactose metabolism (d); and by the intensity of selection (high and low; Figure 3C), where glutamine and glutamate metabolism (a), alanine, aspartate, and glutamate metabolisms (b), arginine and proline metabolisms (c), and galactose metabolism (d) showed greater correlation with protein metabolism.

## 3. Discussion

Generally, genetic selection for growth has been used in cattle breeding programs in order to improve meat production and to increase body weight, average daily gain, hot carcass weight, and *Longissimus* muscle area (LMA) [4,5,6,7]. Based on metabolite concentration, the PLS-DA did not discriminate HG from LG; however, when the permutation and KS test was applied, the findings of this study demonstrated that genetic potential for growth affects muscle energy and protein metabolism. The greater concentration of arginine, carnosine, and leucine in the HG animals could be related to greater muscle deposition in these animals compared to LG, as confirmed by greater LMA by 3 cm^2^ for the HG animals, as described by Silva (unpublish data). Arginine plays an important role in growth metabolism, improving protein synthesis and muscle mass deposition by two biochemical mechanisms: first, by stimulating the activation of the mTOR cell signaling pathway in skeletal muscle [12]; and second, by stimulating the synthesis of a potent vasodilator, nitrogen oxide, which promotes an increase in the blood flow and subsequently the nutrient supply in peripheral tissues [13], thus improving the substrates for protein synthesis and muscle hypertrophy. Additionally, higher leucine levels in HG animals indicate greater hypertrophy for these animals, since leucine plays a key role in the initiation of anabolic processes in muscle [14] and the inhibition of autophagic proteolysis via the mTOR-independent pathway [15], which is an important factor for muscle hypertrophy and protein turnover.

Differences in carnosine levels between HG and LG animals indicates that growth potential (GP) induces a shift in muscle fiber toward the glycolytic type due to the fact that glycolytic fibers have a higher content of imidazole dipeptides such as carnosine than slow oxidative fibers [16]. Supporting this finding, Beline et al. [17] analyzed the muscle fiber types of the same animals as those used in the present study and reported that HG animals showed a higher frequency of fast glycolytic fibers (30.5% vs. 26.9% for HG and LG, respectively), and a lower frequency of slow oxidative fibers (32.6% vs. 39.2% for HG and LG, respectively). These overall changes in muscle fiber composition contribute to changes in muscle metabolism and metabolite concentrations.

Keady et al. [18], studying Angus males, reported that an increase in muscle GP may be associated with increased glycolysis and decreased oxidative metabolism, as there are also greater quantities of mitochondria where the citric acid cycle takes place in slow-twitch compared to fast-twitch muscle fibers, as is evident in lower-muscle-mass animals [18]. In ovine studies, Hamelin et al. [19] reported that the abundance of glycolysis proteins was greater in the *Longissimus* muscle of fast-growing compared to slow-growing rams.

Precocity, or sexual maturity, is also a measurement of great importance to beef cattle producers. The present data shows that genetic potential for precocity can affect muscle protein and fat metabolism. The four most important metabolites, according to VIP score, that differed between the HP and LP animals were glutamine, betaine, creatinine, and isoleucine, all higher in muscle from the HP group. These metabolites are related to muscle protein metabolism, and therefore it can be postulated that improvements in protein deposition in HP cattle may be responsible for their greater muscle growth compared to LP cattle. This is consistent with data reported by Silva (unpublish data), who studied the same animals and reported a LMA greater by 2.1 cm^2^, and heavier retail cuts from carcasses of HP compared to those of LP animals. Therefore, the highlighted pathways are correlated with amino acid metabolism and protein deposition such as glutamine and glutamate metabolism, alanine, aspartate, and glutamate metabolism, arginine and proline metabolism, and purine metabolism. As stated, glutamine is highly correlated with protein metabolism and acts as an anabolic mediator, decreasing protein catabolism and thereby promoting an increase in muscle growth rate. Glutamine and leucine have also been reported by others as being key metabolites in the regulation of muscle protein turnover [20,21] by decreasing protein degradation and/or improving protein synthesis. Similarly, betaine is a metabolite that plays a key role in promoting energy for muscle deposition, providing energy for animal growth [22], and creatinine, as described above, is a muscle mass biomarker [23].

On the other hand, the high concentration of carnitine, acetyl carnitine, and lower levels of glucose in HP animals may indicate differences in fatty acid metabolism. However, according to Silva (unpublish data), no differences in fat deposition were observed between the HP and LP groups, except for rump fat, which was greater in HP animals (5.3 mm vs. 4.7 mm in the HP and LP groups, respectively), suggesting that despite the differences in adipose tissue-specific metabolites, they were not sufficiently different to alter fat deposition in HP carcasses. The lower levels of free carnitine and acetyl carnitine in LP animals may reflect the tissue transport of long-chain fatty acids into the mitochondria for β-oxidation [24]. Alternatively, acetyl carnitine can transfer its acetyl group to the inside of the mitochondria, which can be used in the citric acid cycle for energy generation. Other authors have suggested that a decrease in carnitine levels could be related to its use in promoting β-oxidation, decreasing fatty acid deposition in meat [25]. Lower amounts of muscle glucose in HP animals can be related to its oxidation via the pentose phosphate pathway [26], generating nicotinamide adenine dinucleotide phosphate (NADPH) and promoting lipogenesis. As previously described by Vernon [26], NADPH is required to support lipogenesis, and in ruminants, 50% to 80% of NADPH is produced by glucose oxidation via the pentose phosphate pathway. These results suggest that the animals selected for HP presented changes in muscle metabolism in order to support fat deposition, either by an increase in fat production or a reservation of fat to be used for energy generation by β-oxidation compared to the LP animals.

Additionally, when comparing the four groups (HG, LG, HP, and LP), the results obtained in this work suggest that the intensity of selection (high and low) can affect protein metabolism and muscle hypertrophy regardless of selection trait (growth or precocity), since the HG and HP groups presented similar metabolism patterns with regard to protein metabolism including creatinine, glutamine, fumarate, and isoleucine (biochemical mechanisms explained above), which mostly affect protein metabolisms pathways. On the other hand, the differences between genetic potential for growth and precocity (regardless of high or low intensity) were associated with fat metabolism, according to glycerol and choline concentrations, which mostly affect lipidic pathways. The concentration of these metabolites indicate that animals selected for precocity tend to save fatty acids from oxidation metabolism, showing that HP animals possibly do not use fatty acids as an energy source as HG animals do. 

## 4. Material and Methods

All procedures performed in the present study involving animals were in accordance with the ethical standards of the Institutional Animal Care and Use Committee Guidelines of Faculdade de Zootecnia e Engenharia de Alimentos, Universidade de São Paulo, Protocol No. 8886050916/16.

### 4.1. Post-Weaning Growth Evaluation and Animal Selection

Animals used in this study were from the experimental herd of the University of São Paulo, located in Pirassununga/SP/Brazil and participated in a commercial genetic evaluation program. Animals were born from August to October 2014 and 2015, for years 1 and 2, respectively. All animals were kept on pasture (*Brachiaria decumbens*) from birth up to approximately 20 months of age. The EPDs were obtained using a multi-trait animal model (BLUP). The post-weaning growth EPD was obtained using information of weaning weight (*n* = 494,652) and post-weaning weight gain (211,005). Pedigree data included all animals (*n* = 583,406) with an observation including parents and grandparents. Precocity EPD was obtained using a multi-trait animal model involving information of a 14-month pregnancy rate (*n* = 32,859). Pedigree data included all animals (*n* = 344,023) with an observation including parents and grandparents.

In the first year, 50 animals were selected from a group of 250 young bulls, according to their EPD value for post-weaning growth (GP; growth potential study) and divided into two groups: high GP (HG) and low GP (LG). In the second year, another 50 animals were selected from a group of 183 young bulls, according to their EPD value for precocity (P) and divided in two groups: high P (HP) and low P (LP). After the measurement of traits for the genetic evaluation program, animals were kept in the pasture system until they were transported to the feedlot facilities for finishing.

### 4.2. Finishing, Slaughter, and Carcass Samples

Muscle samples used in this study were collected from a larger project evaluating the effects of EPD groups on animal performance, carcass, and meat quality traits (Silva, unpublish data). Animals were feedlot fed on a basic diet (27:73 forage/concentrate) for approximately 100 days and then slaughtered, according to Humanitarian Slaughter Guidelines as required by Brazilian law; all procedures were the same as those used in commercial conditions. After 24 h of chilling, carcasses were fabricated and *Longissimus lumborum* (LL) muscle samples (10 g) were taken from each animal at the 12th rib level and stored at −80 °C for further analysis of metabolite profile by ^1^H-NMR spectroscopy.

### 4.3. Extraction of Polar Beef Metabolites

A total of 0.5 g of frozen muscle was macerated and homogenized (ULTRA-TURRAX^®^ T25 digital, IKA, Campinas, SP, Brazil) and the metabolites were extracted with 3.5 mL of cold methanol/water solution (4:3 v/v) while vortexing for 1 min, as previously described by Beckonert et al. [9]. Samples were left on ice for 15 min and then centrifuged for 15 min at 10,000× *g* at 4 °C to remove precipitated protein and connective tissue. Supernatants were carefully transferred to Eppendorf tubes and freeze-dried (Itasul Import and Instrumental Technical Ltd., Porto Alegre, RS, Brazil). Remaining residues were reconstituted in 600 μL 100 mM phosphate buffer (containing 10% D_2_O and 90% H_2_O, pH 7.0) and 60 μL internal standard solution (containing 5 mM 3-(trimethylsilyl)-1-propanesulfonic acid sodium salt (DSS)) as a quantitation standard and chemical shift reference, and 100 mM imidazole as a pH indicator). Samples were centrifuged at 10,000 × *g* for 3 min at 4 °C to remove any precipitate. A volume of 600 μL of the supernatant was transferred to standard 5 × 178 mm thin-walled NMR tubes (VWR International).

### 4.4. Nuclear Magnetic Ressonance (NMR) Spectroscopy

One-dimensional proton nuclear magnetic resonance (1D ^1^H-NMR) was used for metabolite profiling. ^1^H-NMR spectra were acquired at 300 K on a Bruker Avance 14.1 T spectrometer (Bruker Corporation, Karlsruhe, Baden-Württemberg, Germany) at 600.13 MHz for ^1^H, using a broad band observe 5 mm probe. D_2_O was used as a lock solvent and DSS as the chemical shift reference for ^1^H. Standard spectra were acquired using a single 90° pulse and each spectrum was the sum of 64 free induction decays. Water suppression was performed using the Bruker “zgesgp” pulse sequence (excitation sculpting with gradients) and the following additional acquisition parameters were used: 13.05 μs 90-degree pulse, 5.0 s relaxation delay, 64 K data points, 64 scans, 3.89 s acquisition time, and 14.03 ppm spectral width.

### 4.5. Spectral Processing and Metabolite Quantitation

1D ^1^H-NMR spectra were processed using Chenomx NMR Suite Professional 7.7 software (Chenomx Inc., Edmonton, Canada): phasing and baseline correction was performed and the pH calibrated using the resonances of imidazole. Spectra were referenced to the DSS methyl peak at 0.00 ppm for quantification of all metabolites. The same peak was also used as a chemical shape indicator (i.e., as an internal standard for quantitation).

Thirty-one metabolites were quantified in the 1D ^1^H-NMR spectra of muscle extracts using the Profiler module on the Chenomx NMR Suite Professional software with an in-built 1D spectral library. Quantitation was by comparing the area of selected metabolite peaks with the area under the DSS methyl peak, which corresponded to a known concentration of 0.5 mM in each sample. The resulting metabolite concentration table was exported to Excel where sample identifiers were added.

### 4.6. Data Analysis

Metabolomic data were analyzed using MetaboAnalyst 4.0 [27]. The metabolite concentration table was uploaded to MetaboAnalyst, and data were log-transformed and Pareto-scaled before analysis. The multivariate approach of partial least squares discriminant analysis (PLS-DA) was applied. Cross validation was performed using the leave-one-out cross-validation method and the performance measure ‘accuracy’. The R2 and Q2 values were used to evaluate the fit and prediction power, respectively, of the PLS-DA model. Due the low Q2 values for PLS-DA validation of growth analysis, the asymptotic general independence test from the “coin” library version 1.3-1 [28] and two-sample Kolmogorov–Smirnov tests were carried out using R version 3.6.1, in order to rank metabolites to discriminate HG and LG. Significance was declared at *p* ≤ 0.05. The analysis for precocity group, follow the PLS-DA model, and a VIP (variable importance in the projection) plot was used to rank the metabolites based on their importance in discriminating groups. Metabolites with the highest VIP values are the most powerful group discriminators. Typically, VIP values >1 are significant and VIP values >2 are highly significant. Furthermore, the important metabolites classified though the VIP score were used to construct the metabolic pathway according to Xia and Wishart [29] using MetaboAnalyst. The compound names were standardized according to the Kyoto Encyclopedia of Genes and Genomes ID, the pathway algorithms used were global test and relative-betweenness centrality, and the library chosen was Bos Taurus (81). Based on the exploratory nature of this study, we included pathways with a raw *p*-value of <0.1 as being of high pathway impact and interest. 

## 5. Conclusions

This study provides greater insight into differential cattle muscle metabolomics across animals divergently selected for growth and precocity, and shows for the first time that predominant metabolic pathways differ greatly between treatments. Growth groups were differentiated by muscle protein and energy metabolites/metabolism, and precocity groups by muscle protein and fat metabolites/metabolism. Both high growth and high precocity appear to be correlated with protein metabolism, protein metabolites and protein pathways being highlighted when compared to low growth and low precocity, while the selection for precocity correlated with changes in fat metabolism including metabolites and related pathways when compared to animals selected for growth.

## Figures and Tables

**Table 1:** Asymptotic general independence test (Permutation) and two-sample Kolmogorov–Smirnov test (KS) applied for muscle metabolites in animals with genetic potential for growth.

Metabolites	*p*-Value	
	Permutation	KS
Isoleucine	0.4675185	0.07832309 (D = 0.36)
Valine	0.178246	0.6993742 (D = 0.2)
Lactate	0.07011515	0.1545381 (D = 0.32)
Beta-Alanine	0.5255741	0.6993742 (D = 0.2)
Proline	0.406171	0.28096 (D = 0.28)
Succinate	0.1662221	0.6993742 (D = 0.2)
Methionine	0.849174	0.6993742 (D = 0.2)
Glutamine	0.7481134	0.6993742 (D = 0.2)
Glutamate	0.04236974	0.28096 (D = 0.28)
Creatine	0.06499233	0.1545381 (D = 0.32)
Creatinine	0.1402015	0.4675586 (D = 0.24)
Alanine/Hypoxantine	0.1040305	0.28096 (D = 0.28)
Glycerol	0.474837	0.07832309 (D = 0.36)
Leucine	0.0316985	0.03663105 (D = 0.4)
Threonine	0.9695724	0.9937649 (D = 0.12)
Choline	0.3409856	0.4675586 (D = 0.24)
Glucose	0.154457	0.28096 (D = 0.28)
Arginine	0.000686095	0.002318458 (D = 0.52)
Carnosine	0.002277323	0.03663105 (D = 0.4)
Carnitine	0.8327426	0.9062064 (D = 0.16)
Acetylcarnitine	0.3393883	0.9062064 (D = 0.16)
Adenine	0.9329617	0.9937649 (D = 0.12)
Inosine	0.6008663	0.1545381 (D = 0.32)
Betaine	0.8918807	0.9937649 (D = 0.12)
Fumarate	0.5587293	0.9062064 (D = 0.16)
Glycerate	0.6074683	0.6993742 (D = 0.2)
Anserine	0.1455512	0.6993742 (D = 0.2)
NADH	0.3460372	0.4675586 (D = 0.24)
IMP	0.05199825	0.1545381 (D = 0.32)
ATP	0.7473122	0.4675586 (D = 0.24)
Fructose	0.6223233	0.6993742 (D = 0.2)

**Table 2:** Results from the polar meat extract pathway analysis from comparisons between HP and LP, Growth and Precocity (regardless the intensity of selection) and High and Low (regardless of selection trait).

Metabolic Pathway	Total Cmpd	Hits	Raw *p*	−log(*p*)	Impact
*HP and LP*					
Alanine, aspartate and glutamate metabolism	23	4	5.01 × 10^−7^	14.508	0.4033
Purine metabolism	68	4	1.01 × 10^−6^	13.803	0.1290
D-Glutamine and D-glutamate metabolism	5	2	1.33 × 10^−6^	13.533	1
Arginine and proline metabolism	44	5	2.27 × 10^−6^	12.994	0.1883
*Growth and Precocity*					
Glycine, serine and threonine metabolism	32	3	1.58 × 10^−56^	128.49	0.2919
Glycerophospholipid metabolism	29	1	2.59 × 10^−42^	95.758	0.0244
Glycerolipid metabolism	18	1	2.52 × 10^−29^	65.85	0.2809
Galactose metabolism	26	2	4.43 × 10^−28^	62.984	0.0364
*High and Low*					
Alanine, aspartate and glutamate metabolism	23	4	3.34 × 10^−61^	139.25	0.4033
Arginine and proline metabolism	44	4	9.38 × 10^−60^	135.92	0.1772
D-Glutamine and D-glutamate metabolism	5	2	2.56 × 10^−56^	128.01	1
Nitrogen metabolism	9	3	1.95 × 10^−51^	116.76	0
Galactose metabolism	26	2	7.87 × 10^−51^	115.37	0.0364

Total Cmpd: the total number of compounds in the pathway; Hits: the actually matched number from the user uploaded data; Raw p: the original P-value calculated from the enrichment analysis; Impact: the pathway impact value calculated from pathway topology analysis.

## Supplementary Materials

The following are available online at https://www.mdpi.com/2218-1989/10/2/58/s1, Table S1: Descriptive analysis of metabolites concentrations (mg/g of fresh meat). Figure S1: Validation statistics for PLS-DA on animals selected for growth. Figure S2: Validation statistics for PLS-DA on animals selected for precocity.
