# Supplementary material for: Selection for Growth and Precocity Alters Muscle Metabolism in Nellore Cattle
Source: Metabolites. 2020 Feb 6;10(2):58. doi: 10.3390/metabo10020058 (PMC7073857; doi:10.3390/metabo10020058)
Supplement: Supplementary file 1 [file metabolites-10-00058-s001.pdf]

Supplementary material

Table S1. Descriptive analysis of metabolites concentrations (mg/g of fresh meat).

Metabolites	Growth[1]		SEM	Precocity[2]		SEM
	HG	LG		HP	LP	
Lactate	44.83	54.64	2.709	50.38	48.79	3.120
Creatine	20.43	23.50	0.832	14.66	19.15	0.020
Carnosine	12.45	8.64	0.624	12.17	12.27	0.131
Arginine	12.25	8.12	0.608	7.09	9.50	0.661
Glucose	3.10	3.81	0.250	3.21	3.94	0.213
Glycerate	2.25	2.41	0.153	2.72	1.74	0.030
Glycerol	2.18	1.91	0.190	1.10	1.59	0.045
Glutamine	2.00	1.85	0.139	2.51	0.68	0.103
Carnitine	1.65	1.69	0.105	0.93	0.63	0.092
Creatinine	1.56	1.09	0.115	1.22	0.70	1.260
Leucine	1.11	0.86	0.058	0.90	0.60	0.089
Betaine	0.96	0.98	0.072	0.13	0.07	0.008
Methionine	0.91	0.88	0.071	0.06	0.05	0.005
Choline	0.82	1.19	0.054	0.56	0.45	0.142
Acetyl carnitine	0.70	0.84	0.072	0.56	0.35	0.048
ATP	0.68	0.62	0.088	0.54	0.49	0.098
Threonine	0.67	0.67	0.042	0.69	0.47	0.050
Alanine	0.62	0.48	0.043	0.43	0.41	0.091
IMP	0.59	0.88	0.073	0.52	0.42	0.038
Adenine	0.49	0.50	0.036	0.39	0.24	0.036
Anserine	0.47	0.63	0.056	0.93	0.66	0.071
Inosine	0.46	0.49	0.029	0.79	0.63	0.075
Glutamate	0.35	0.66	0.028	0.54	0.82	0.136
Fructose	0.33	0.35	0.028	0.32	0.29	0.022
NADPH	0.26	0.30	0.023	0.45	0.27	0.043
Valine	0.22	0.28	0.022	0.35	0.22	0.044
Isoleucine	0.16	0.15	0.013	0.19	0.13	0.022
B-Alanine	0.14	0.15	0.009	0.11	0.18	0.013
Fumarate	0.25	0.10	0.009	0.34	0.18	0.224
Proline	0.21	0.19	0.010	0.14	0.9	0.182
Succinate	0.05	0.06	0.003	0.33	0.15	0.035

[1] Genetic potential for post-weaning growth: high growth (HG) and low growth (LG).

[2] Genetic potential for post-weaning precocity: high precocity (HP) and low precocity (LP).

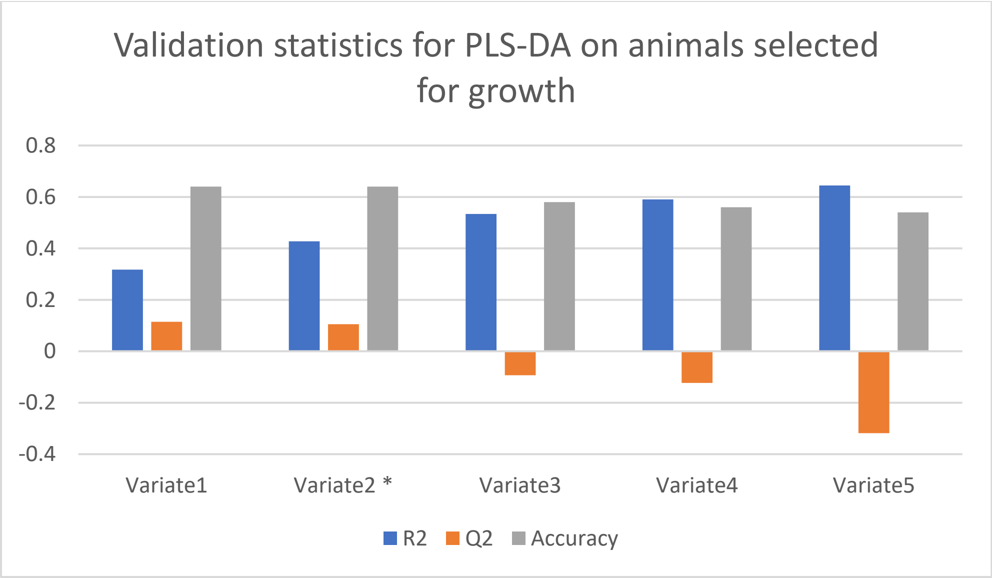

Figure S1: Validation statistics for PLS-DA on animals selected for growth.

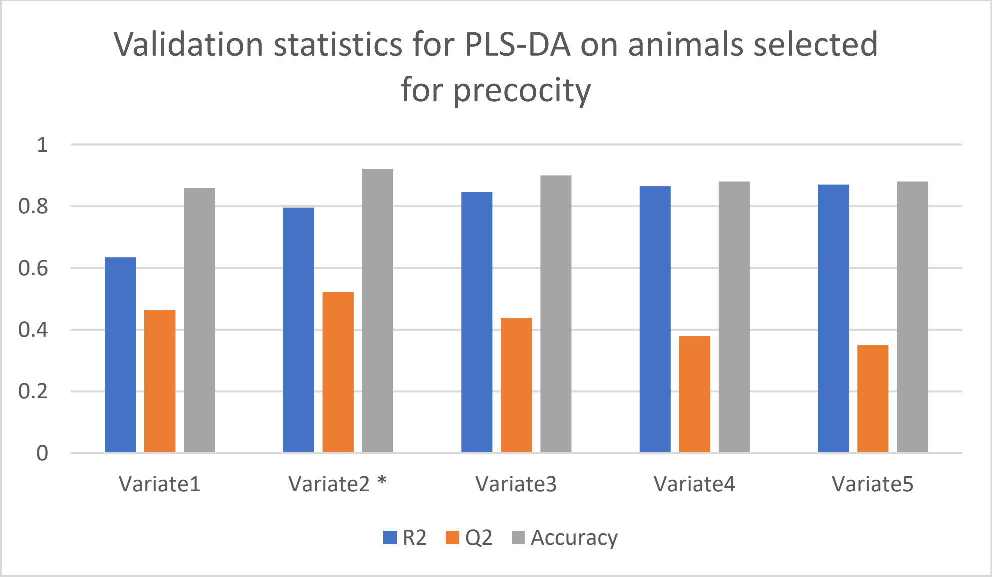

Figure S2: Validation statistics for PLS-DA on animals selected for precocity.
